# Supplementary material for: Air Quality Monitoring Using Low-Cost Sensors in Urban Areas of Jodhpur, Rajasthan
Source: Int J Environ Res Public Health. 2024 May 14;21(5):623. doi: 10.3390/ijerph21050623 (PMC11120845; doi:10.3390/ijerph21050623)

Supplementary Figure S1: PM2.5, | Low Income Area (INDOOR & OUTDOOR) [1]

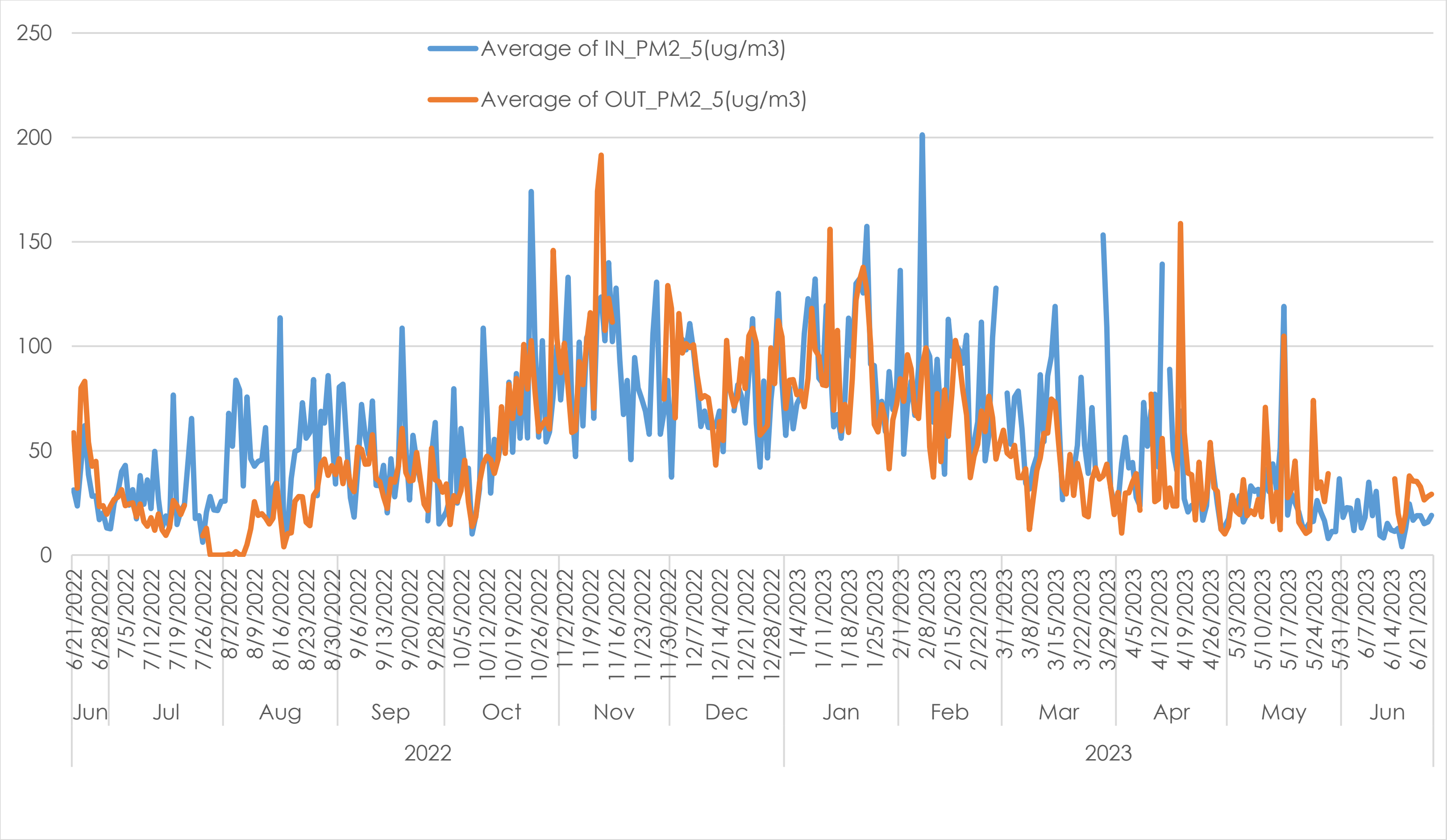

Supplementary Figure S2: PM2.5, | Low Income Area (INDOOR & OUTDOOR) [2]

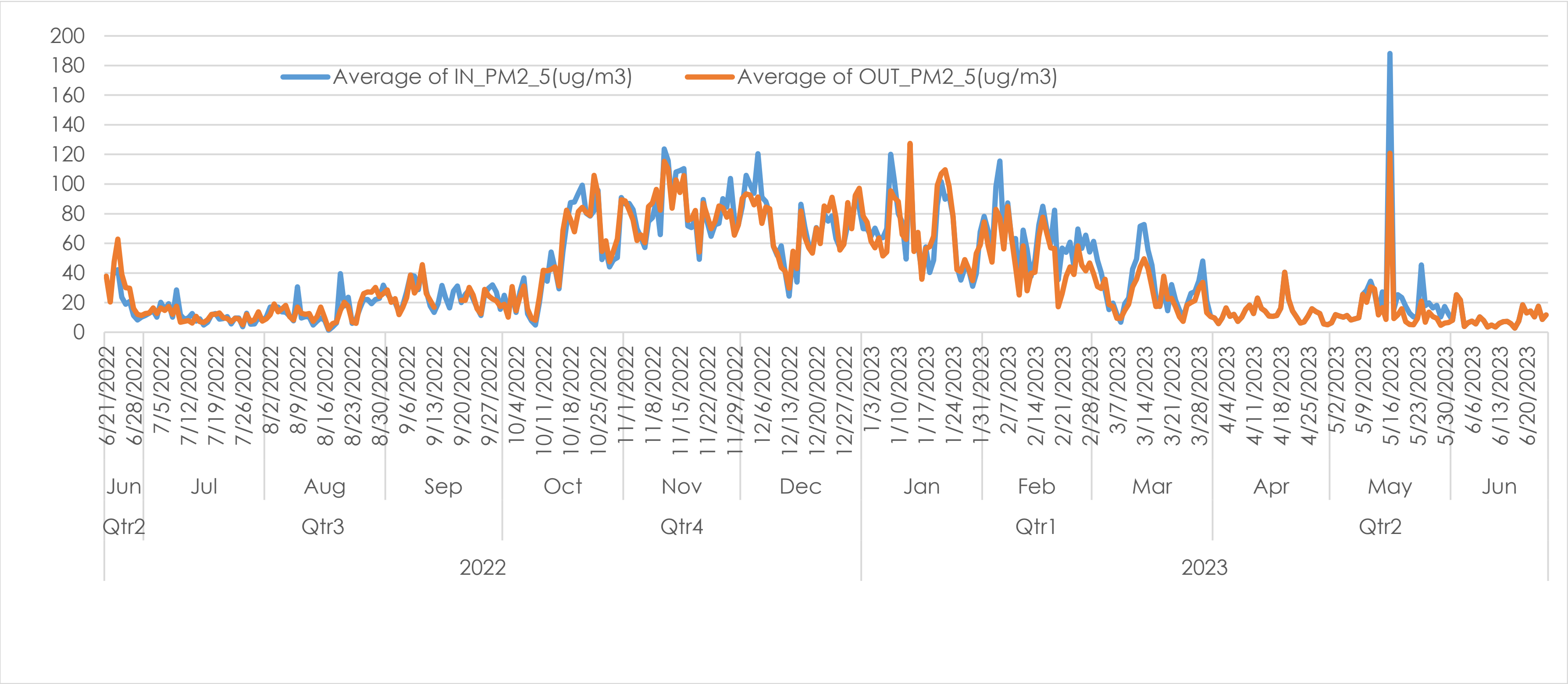

Supplementary Figure S3: PM2.5| Industrial Area (INDOOR & OUTDOOR)-[1]

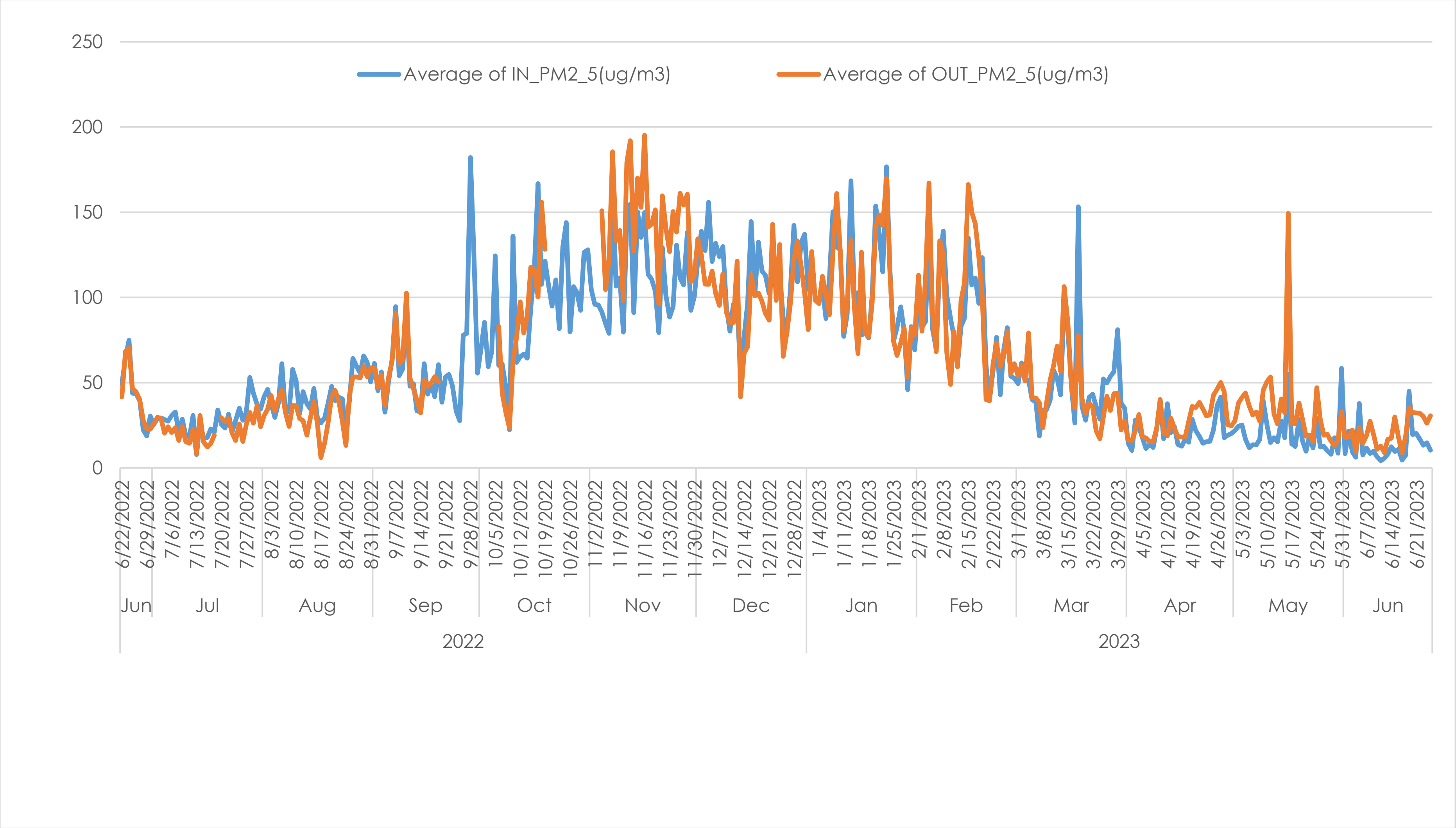

Supplementary Figure S4: PM2.5| Industrial Area (INDOOR & OUTDOOR)-[2]

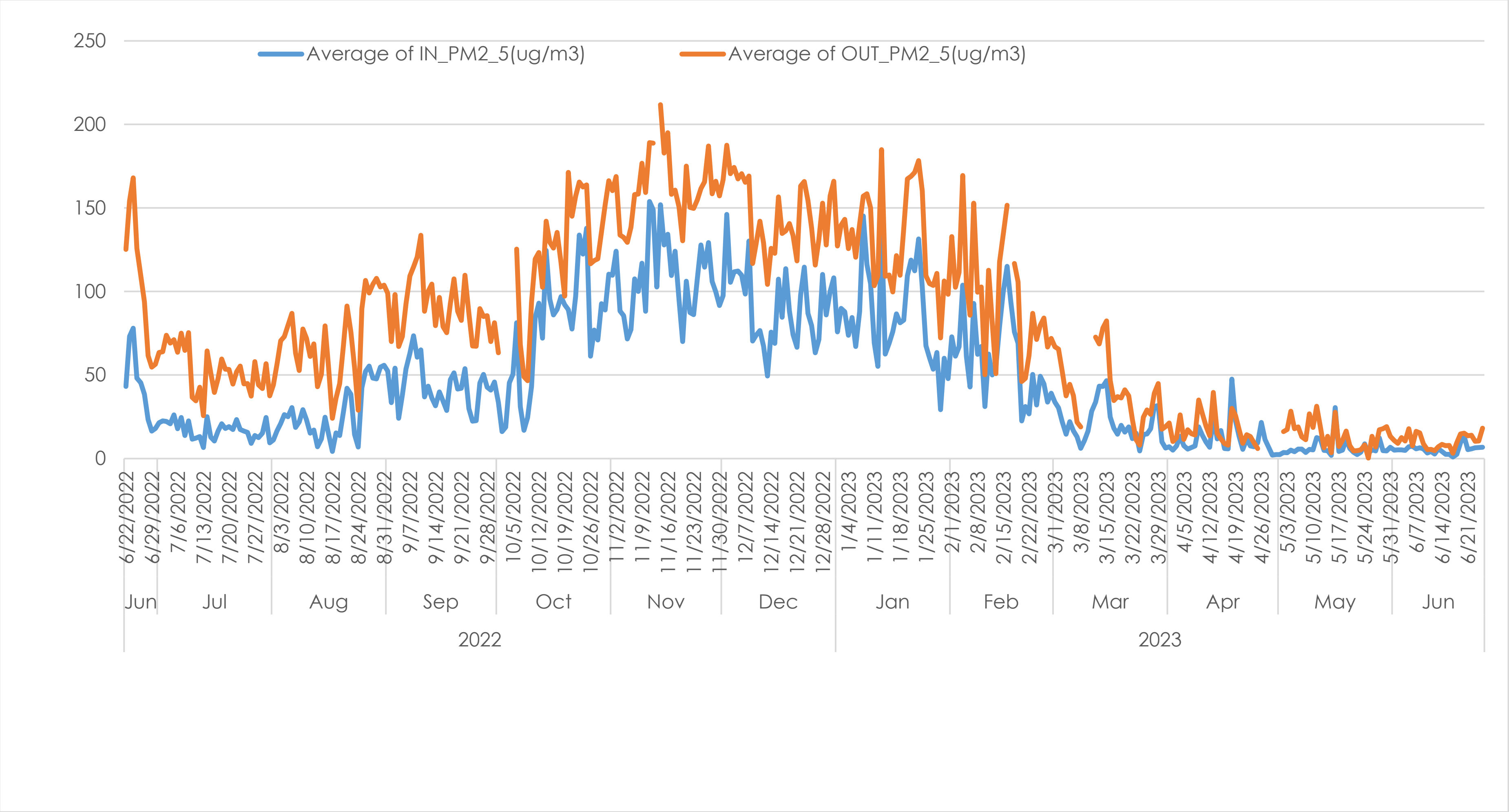

Supplementary Figure S5: PM2.5|Commercial Area (INDOOR & OUTDOOR) [1]

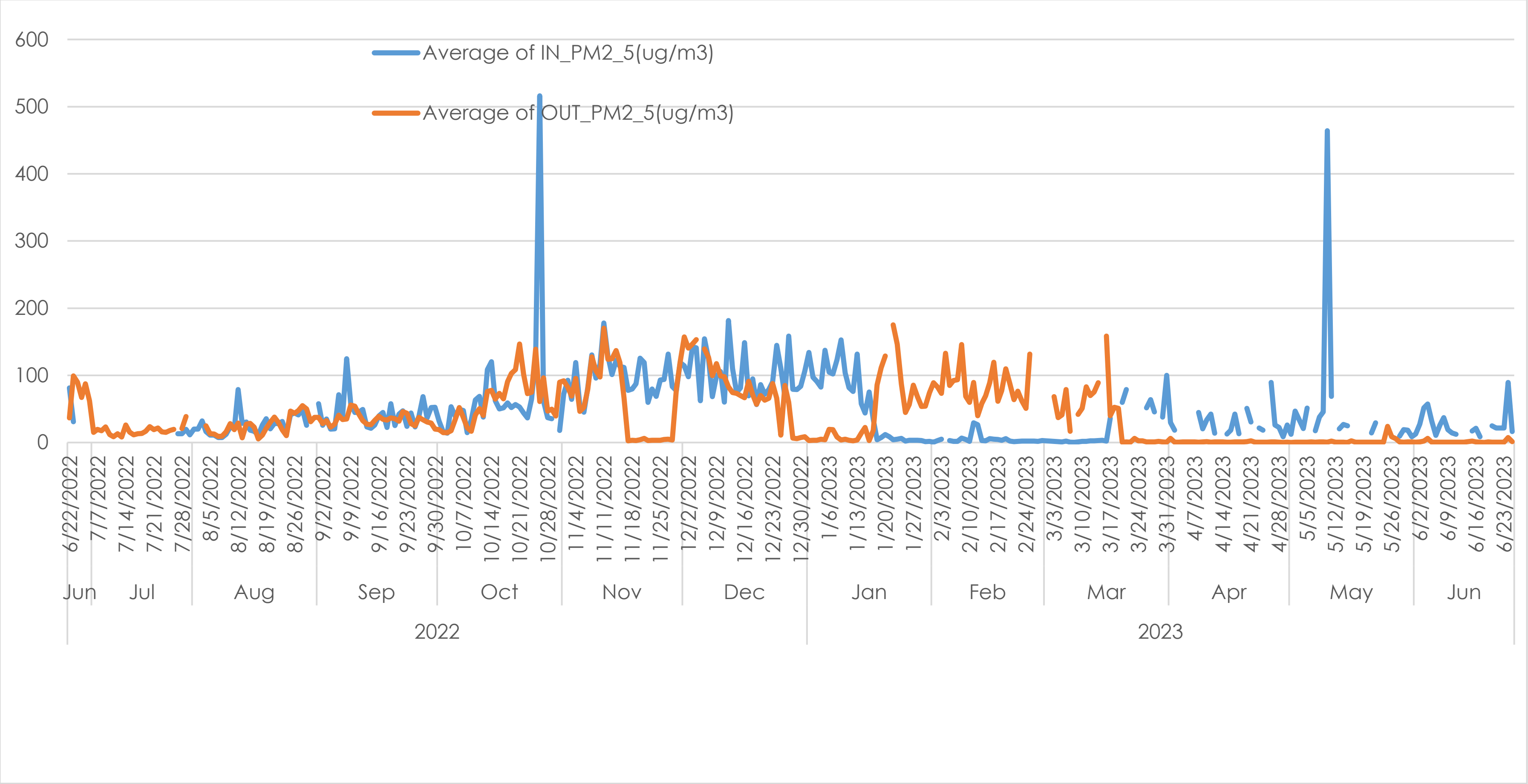

Supplementary Figure S6: PM2.5|Commercial Area (INDOOR & OUTDOOR) [2]

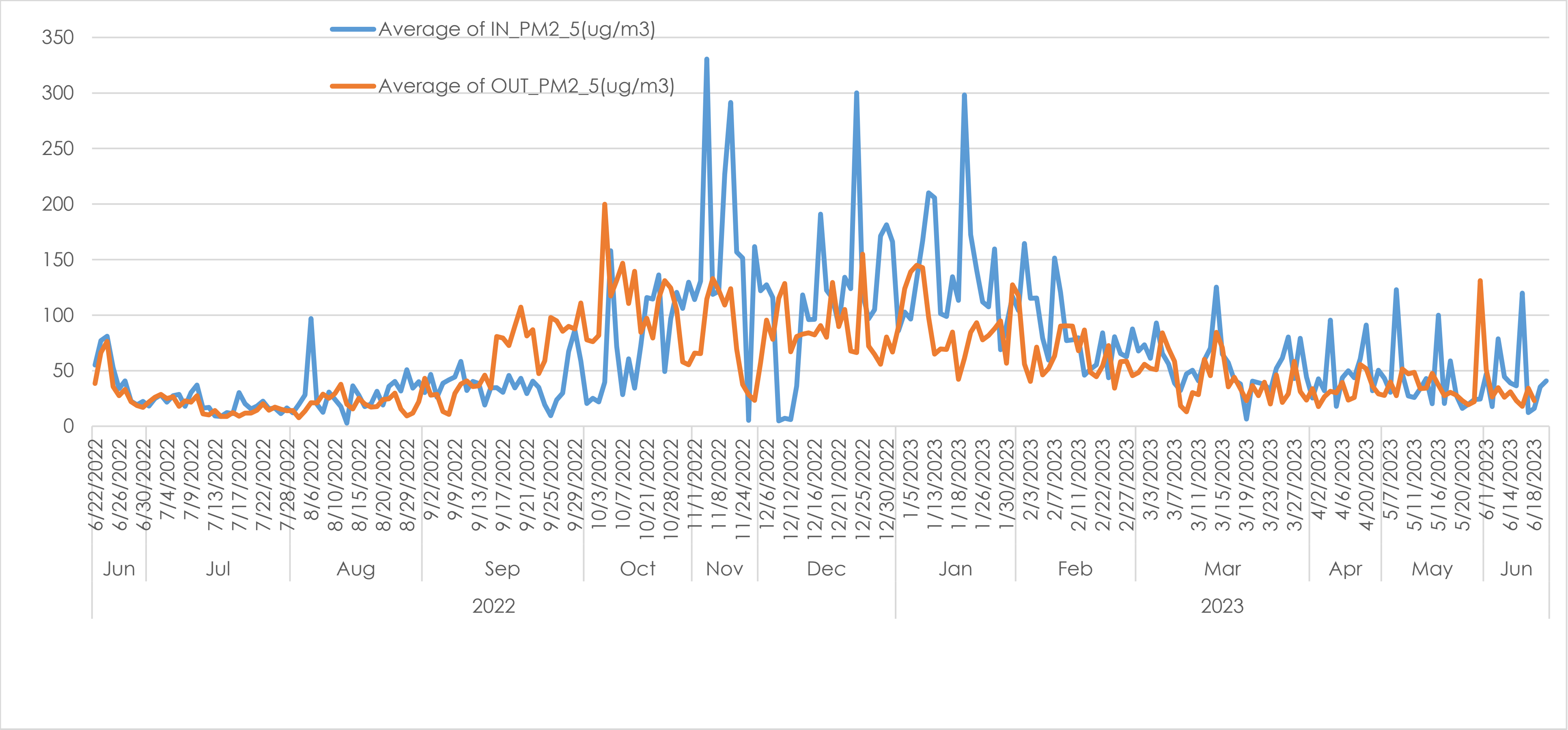

Supplementary Figure S7: PM2.5| High Income Area (INDOOR & OUTDOOR) [1]

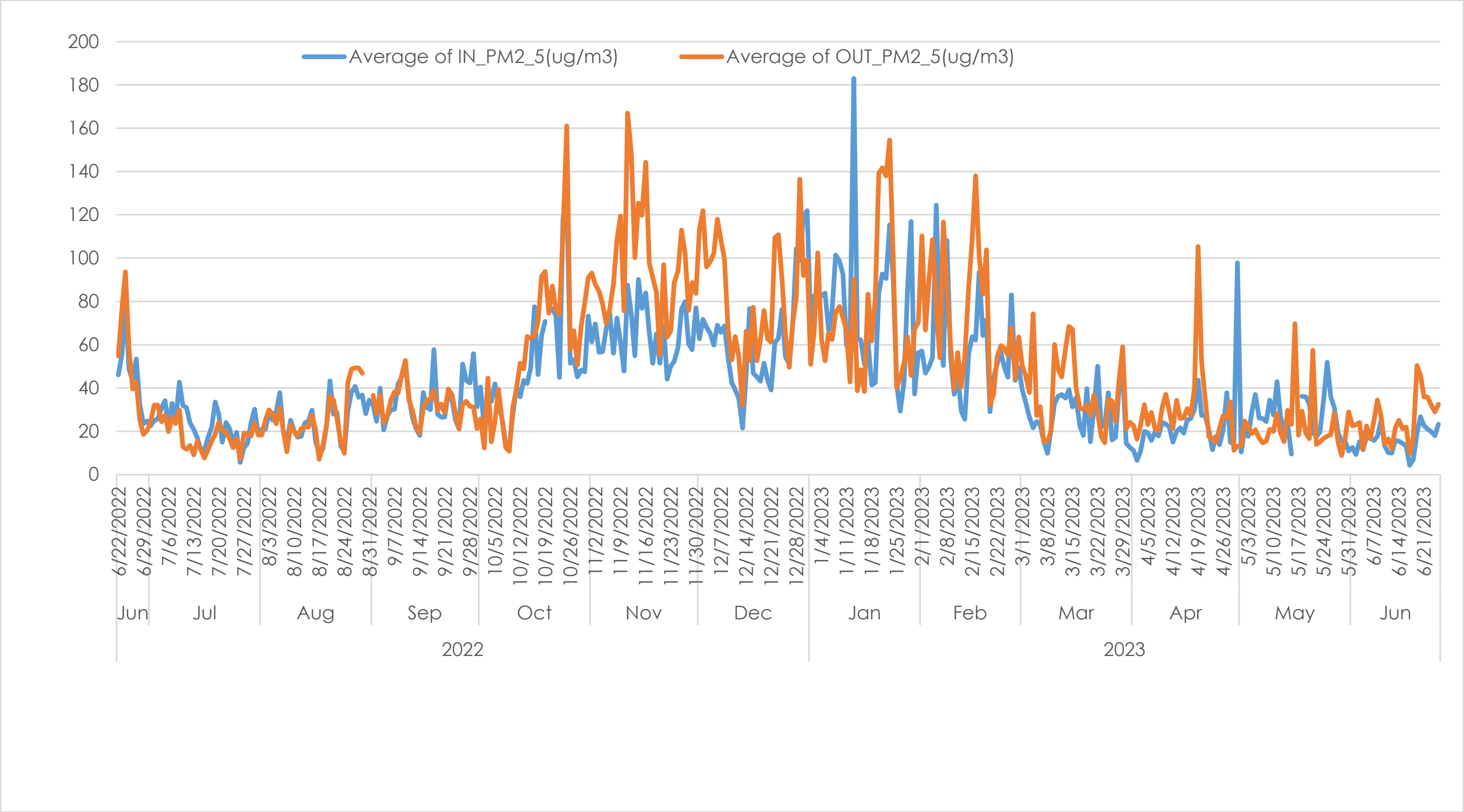

Supplementary Figure S8: PM2.5| High Income Area (INDOOR & OUTDOOR) [2]

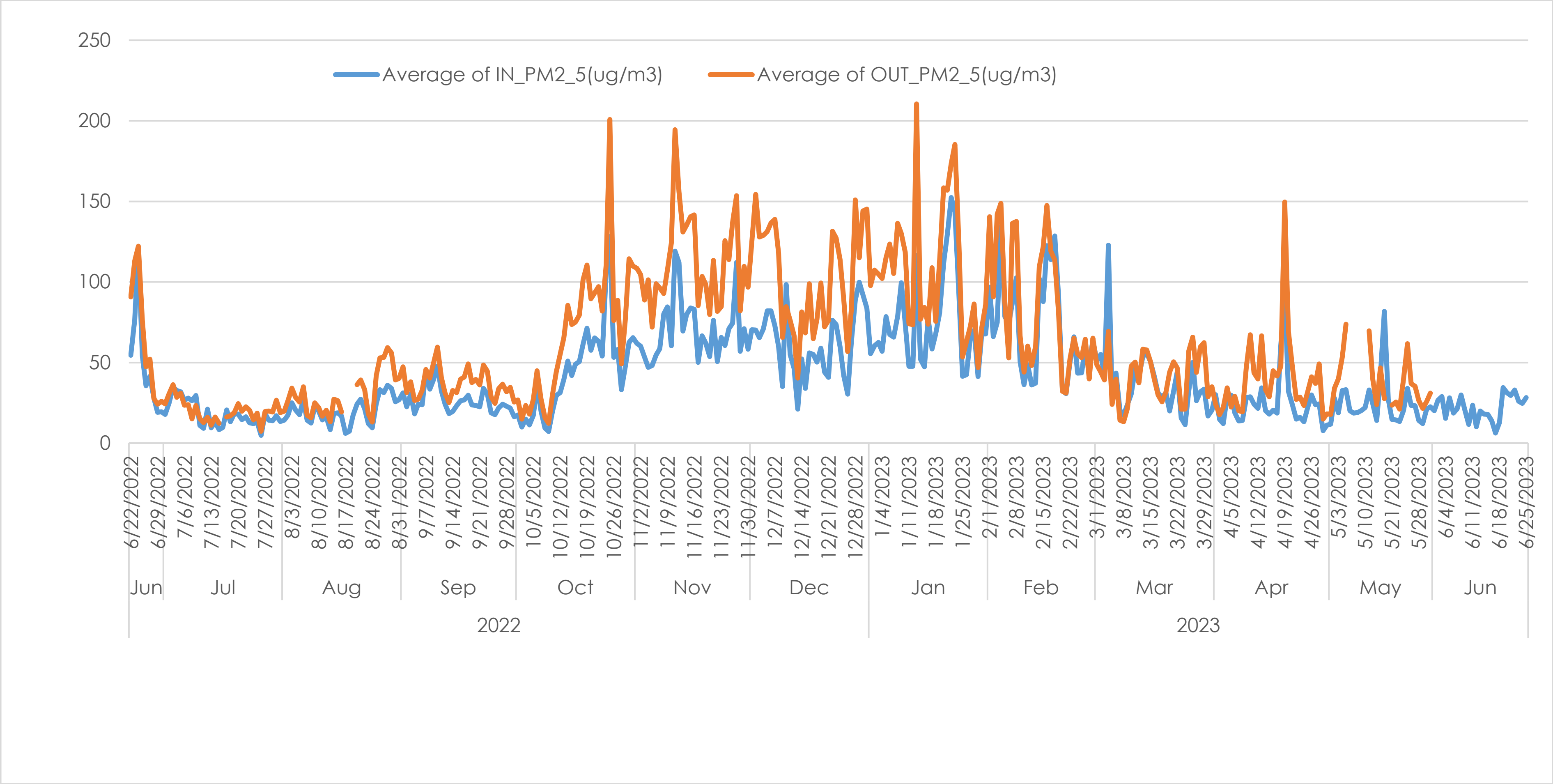

Supplementary Figure S9: PM2.5| Slum Area (INDOOR & OUTDOOR) [1]

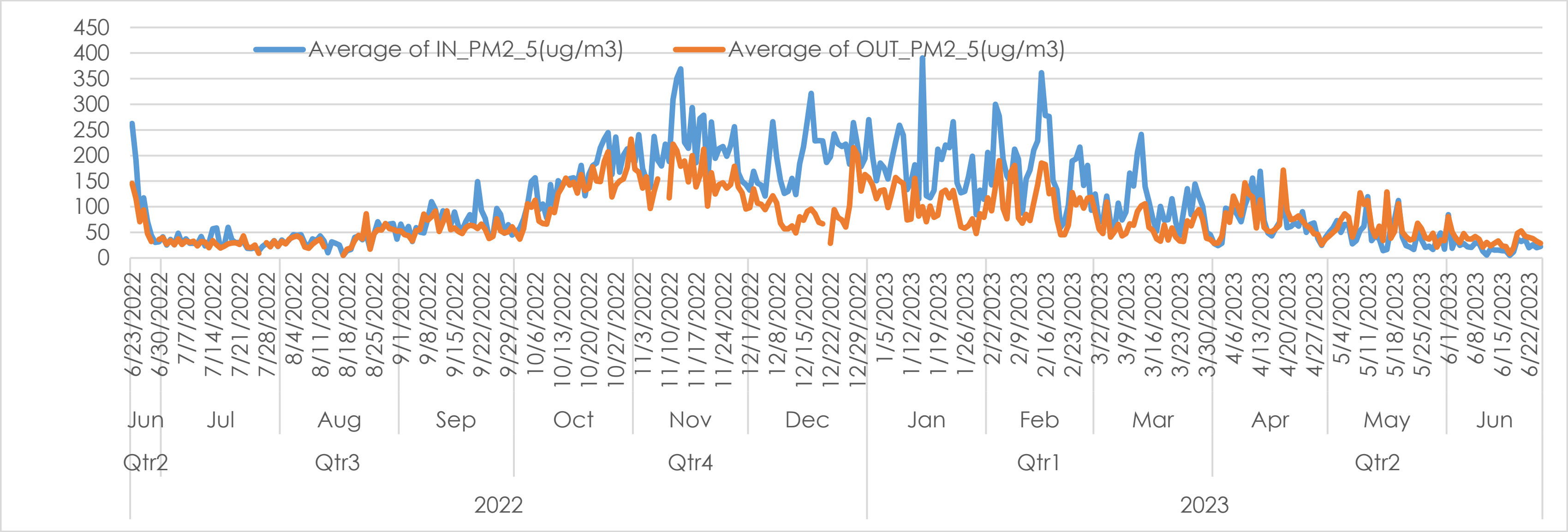

Supplementary Figure S10: PM2.5| Slum Area (INDOOR & OUTDOOR) [2]

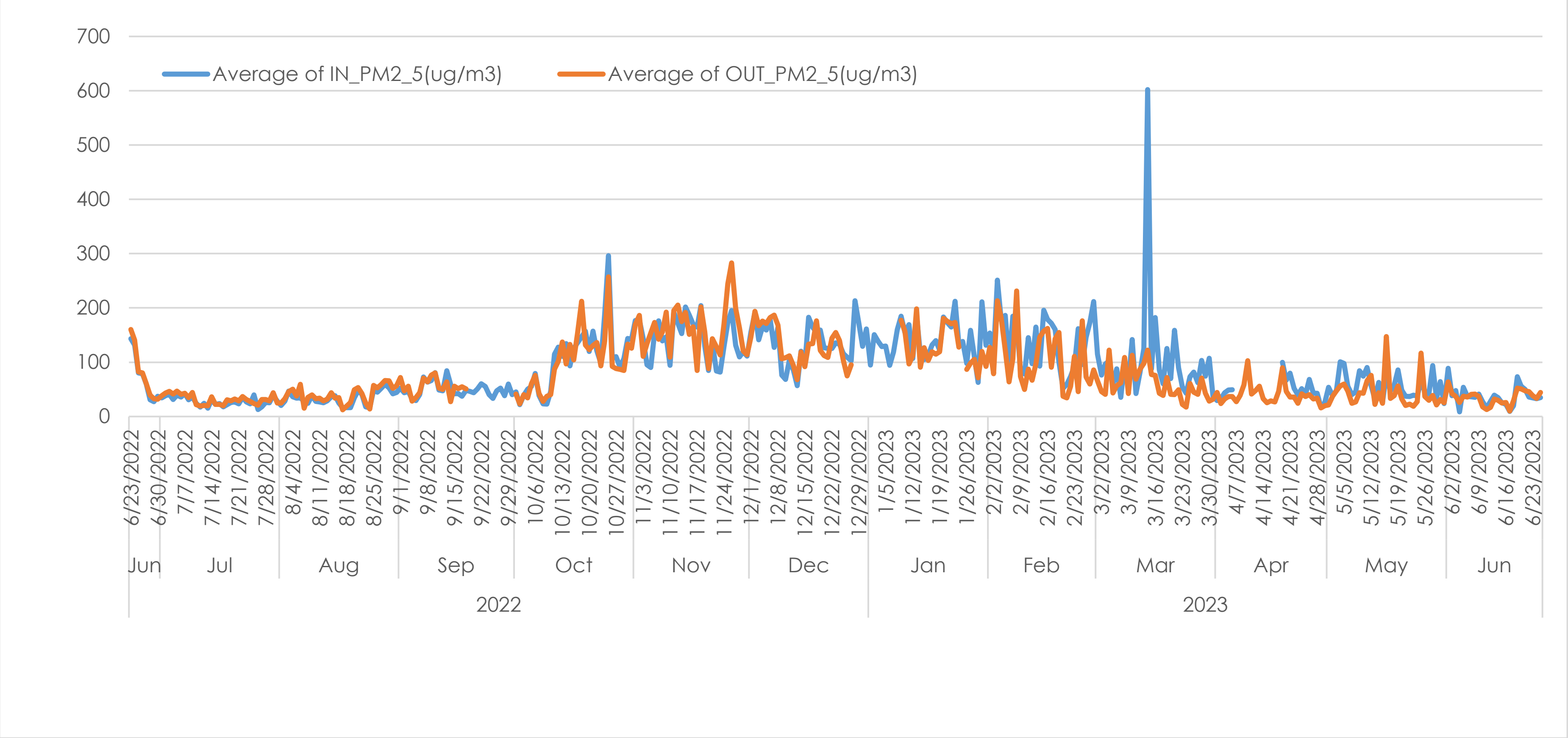

Supplementary Figure S11: PM2.5| Middle Income Area (INDOOR & OUTDOOR) [1]

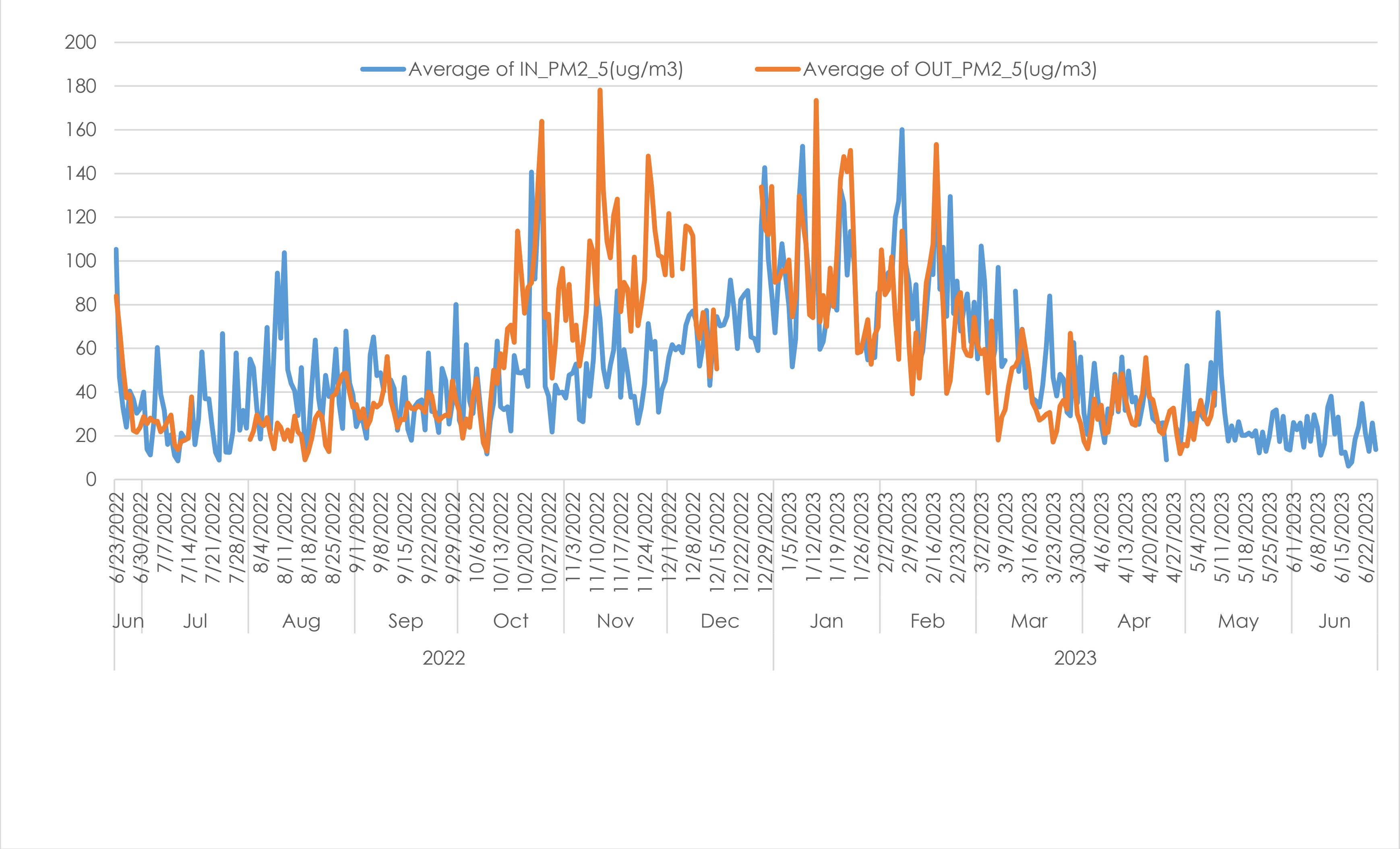

Supplementary Figure S12: PM2.5| Middle Income Area (INDOOR & OUTDOOR) [2]

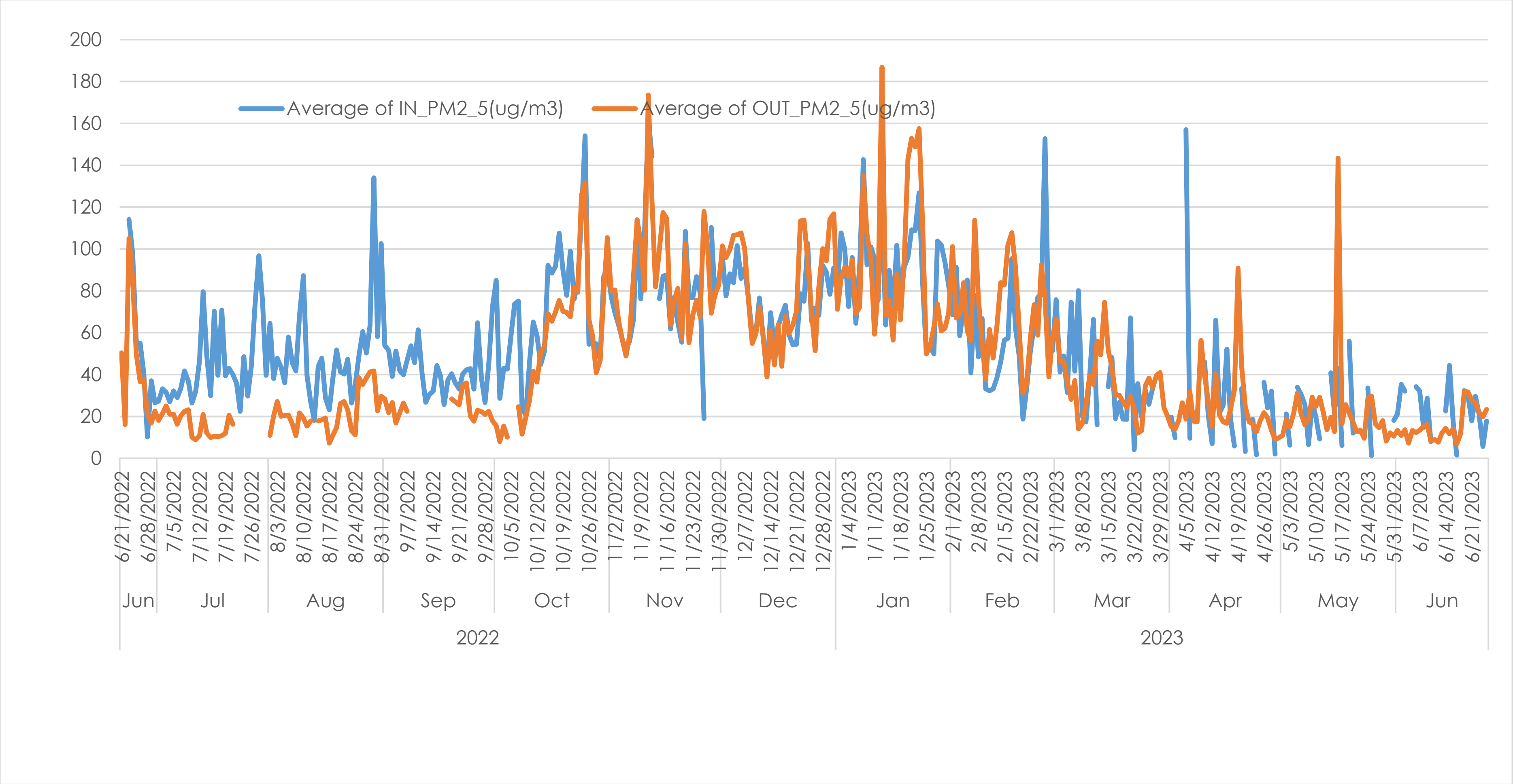

Supplement: Supplementary file 1 [file ijerph-21-00623-s001.zip › ijerph-2955231-supplementary-figures-conv.pdf]
