# Supplementary material for: Air Quality Monitoring Using Low-Cost Sensors in Urban Areas of Jodhpur, Rajasthan
Source: Int J Environ Res Public Health. 2024 May 14;21(5):623. doi: 10.3390/ijerph21050623 (PMC11120845; doi:10.3390/ijerph21050623)
Supplement: Supplementary file 1 [file ijerph-21-00623-s001.zip › ijerph-2955231-supplementary-tables-conv.pdf]

Table S1. Monthly average $PM_{2.5}$ values at all sites (Where i=indoor, o=outdoor, 1=first house, 2=second house).

Location	Site	2022						2023								
		Jul	Aug	Sep	Oct	Nov	Dec	Jan	Feb	Mar	Apr	May	Jun	Minimum	Maximum	Mean ± Standard Deviation
Industrial area	1i	54.83	46.53	55.57	72.61	106.5	112.61	113.14	104.64	96.43	71.01	53.16	67.08	46.53	113.14	79.51±25.42
	1o	47.83	33.35	43.4	59.24	94.87	144.62	102.35	104.06	96.65	58.93	48.73	57.99	33.35	144.62	74.34±33.38
	2i	52.79	32.59	43.76	48.99	83.83	108.83	91.64	84.46	75.65	47.05	51.57	63.05	32.59	108.83	65.35±23.14
	2o	58.91	31.09	37.68	42.66	80.73	103.09	79.16	68.19	78.5	47.16	45.03	49.43	31.09	103.09	60.14±21.74
Commercial area	1i	40.95	33.54	50.41	57.8	73.02	99.62	97.9	99	50.38	65.87	43.5	67.42	33.54	99.62	64.95±23.39
	1o	72.83	28.97	39.51	38.57	71.97	104.75	98.13	84.52	87.58	67.11	38.11	77.99	28.97	104.75	67.50±25.49
	2i	33.09	44.14	40.79	90.17	197.6	136.89	131.2	85	64.07	56.14	66.97	53.16	33.09	197.55	83.26±49.00
	2o	29.52	45.96		76.06	101.6	100.91	90.19	73.93	56.36	43.05	49.99	36.78	29.52	101.64	64.04±25.80
Slum area	1i	50.08	51.88	76.77	162.6	214.7	194.67	170.73	187.61	108.37	85.44	70.52	72.92	50.08	214.73	120.53±60.98
	1o	38.88	49.28	62.27	135.1	155.1	101.61	103.54	116.04	72.09	84.75	68.62	63.61	38.88	155.10	87.58±35.34
	2i	42.98	46.63	57.11	111.8	140.6	133.11	136.22	147.33	102.5	62.1	70.51	66.27	42.98	147.33	93.10±39.61
	2o	40.88	49.53	56.48	115.3	162.6	132.36	127.39	119.97	73.83	58.32	59.88	61.94	40.88	162.58	88.20±40.55
High-income Colony	1i	48.44	45.13	47.9	79.36	75.04	70.07	82.69	76.56	71.32	75.01	44.7	56	44.7	82.69	64.35±14.68
	1o	29.95	36.05	36.33	71.6	99.37	83.42	78.95	83.22	50.66	46.11	58.52	45.71	29.95	99.37	59.99±22.67
	2i	32.12	30.79	34.25	59.44	71.83	66.63	75.36	80.61	54.65	49.13	47.08	47.5	30.79	80.61	54.12±17.04
	2o	29.79	38.56	40.14	80.29	114.4	103.13	106.88	97.37	55.37	53.11	51.53	69.19	29.79	114.36	69.98±29.57
Middle-income Colony	1i	63.41	80.32	58.35	81.18	65.07	80.48	88.02	96.77	64.64	48.41	49.28	59.62	48.41	96.77	69.63±15.42
	1o	40.09	35.59	37.4	75.84	99.33	94.05	95.78	83.6	53.35	51.39	42.04	49.37	35.59	99.33	63.15±24.74
	2i	82.88	74.59	56.23	85.78	84.44	76.28	95.27	74.38	63.46	61.51	50.15	52.63	50.15	95.27	71.47±14.51
	2o	31.55	33.28	32.22	75.52	90.94	83.67	93.08	78.85	50.03	48.33	57.32	42.52	31.55	93.08	59.78±23.45
Low-income Colony	1i	69.81	78.34	66.47	77.61	93.2	82.28	95.92	92.81	76	73.15	57.81	45.23	45.23	95.92	75.72±14.88
	1o	34.38	35.54	44.74	65.82	92.47	83.4	88.28	75.75	53.69	65.66	72.86	44.91	34.38	92.47	63.13±20.27
	2i	45.46	37.17	37.79	38.26	65.89	85.35	73.02	68.51	70.42	45.3	46.51	70.68	37.17	85.35	57.03±16.88
	2o	43.84	27.87	28.62	34.82	70.35	84.2	74.28	71.5	68.3	44.89	46.54	65.32	27.87	84.20	55.04±19.43

Table S2. Monthly average Temperature values at all sites (Where i=indoor, o=outdoor, 1=first house, 2=second house).

Location	Site	2022						2023								
		Jul	Aug	Sep	Oct	Nov	Dec	Jan	Feb	Mar	Apr	May	Jun	Minimum	Maximum	Mean ± Standard Deviation
Industrial area	1i	29.15	28.21	29.49	28.2	24.78	19.7	18.24	24.1	27.57	30.09	30.78	30.93	18.24	30.93	26.77±4.22
	1o	30.12	28.62	30.56	28.58	22.92	21.03	18.53	22.78	26.71	30.78	32.9	35.29	18.53	35.29	27.40±5.09

	2i	30.46	30.15	32.18	30.42	25.75	21.46	18.97	24	28.71	32.23	34.26	32.87	18.97	34.26	28.45±4.84
	2o	29.57	29.57	32.03	30.54	25.32	20.69	17.95	25.18	28.3	31.92	34.12	34.07	17.95	34.12	28.27 ±5.10
Commercial area	1i	28.15	29.29	30.83	30.2	27.2	22.58	19.3	26.46	28.66	30.26	30.65	31.31	19.3	31.31	27.90 ±3.64
	1o	29.94	29.02	32.07	30.95	27.01	22.36	19.54	22.77	27.76	32.5	33.26	31.84	19.54	33.26	28.25 ±4.51
	2i	29.71	28.6	29.82	27.5	25.3	20.97	17.99	22.69	26.15	27.7	30.32	30.31	17.99	30.32	26.42 ±4.00
	2o	30.36	28.93			20.67	17.76	22.89	25.84	28.62	32.95	31.29	31.78	17.76	32.95	27.10 ±514
Slum area	1i	30.25	29.23	31.44	30.29	26.47	22.14	18.99	24.32	27.21	30.1	31.97	32.27	18.99	32.27	27.89 ±4.2
	1o	29.37	28.48	30.86	30.16	25.62	21.34	18.03	24.92	27.17	30.92	32.62	33.3	18.03	33.3	27.73 ±4.59
	2i	28.53	28.32	30.12	29.95	26.81	22.78	19.6	25	27.92	30.23	33.43	31.25	19.6	33.43	27.823 ±3.83
	2o	29.31	28.91	31.41	30.28	26.2	21.82	18.68	25.01	27.53	31.3	32.73	32.81	18.68	32.81	28±4.4
High-income Colony	1i	29.5	28.95	30.02	29.14	25.61	20.66	17.73	22.42	26.77	29.48	31.29	30.01	17.73	31.29	26.8±4.33
	1o	28.54	27.66	29.92	28.39	24	19.96	18.15	23.06	25.55	29.48	29.89	32.85	18.15	32.85	26.45±4.4
	2i	30.13	29.24	31.41	30.7	27.33	23.28	20.46	24.36	28.58	31.5	32.12	31.82	20.46	32.12	28.41±3.81
	2o	28.68	28.56	30.95	30.26	25.6	21.66	18.32	24.58	27	31.27	31.68	31.74	18.32	31.74	27.53±4.29
Middle-income Colony	1i	27.18	25.88	26.95	26.66	25.55	23.42	20.69	23.49	27.1	28.29	28.79	28.14	20.69	28.79	26.01±2.39
	1o	28.95	28.47	30.14	28.11	23.87	20.05	16.54	22.83	25.65	29.51	30.33	31.1	16.54	31.1	26.3±4.59
	2i	28.55	28.38	29.15	29.27	27.82	23.83	21.51	24.92	28.03	30.14	29.72	29.68	21.51	30.14	27.58±2.71
	2o	30.35	28.64	31.69	32.07	28.82	23.92	20.88	27.33	29.09	31.56	32.38	31.22	20.88	32.38	29.00±3.52
Low-income Colony	1i	29.72	28.87	30.13	27.68	23.93	19.68	17.36	21.8	26.55	29.22	31.04	31.89	17.36	31.89	26.49±4.73
	1o	30.38	29.25	30.9	28.11	24.93	18.3	15.88	22.67	25.82	30.75	35.67	33.27	15.88	35.67	27.16±5.92
	2i	30.58	29.64	31.56	29.87	24.05	20.76	19.32	22.83	27.32	28.31	31.14	32.4	19.32	32.4	27.32±4.47
	2o	30.19	29.31	30.48	27.68	24.94	19.99	16.54	20.48	24.65	28.3	31.48	32.51	16.54	32.51	26.38±5.1

Table S3. Monthly average Humidity values at all sites (Where i=indoor, o=outdoor, 1=first house, 2=second house).

Location	Site	2022						2023								
		Jul	Aug	Sep	Oct	Nov	Dec	Jan	Feb	Mar	Apr	May	Jun	Minimum	Maximum	Mean ± Standard Deviation
Industrial area	1i	77.75	81.44	70.46	50.83	47.21	46.99	53.9	50.76	48.89	43.12	49.21	58.95	43.12	81.44	56.63±12.84
	1o	76.82	79.99	62.52	43.59	44.16	42.65	51.26	38.01	40.82	32.32	33.74	56.38	32.32	79.99	50.19±15.82
	2i	72.2	76.89	62.51	43.69	42.79	40.61	43.82	44.24	42.35	34.89	43.92	54.82	34.89	76.89	50.23±13.38

	2o	75.41	78.1	63.24	44.45	41.51	40.96	46.82	42.75	42.44	34.92	45.86	54.21	34.92	78.1	50.89±14.04
Commercial area	1i	81.11	77.79	67.74	50.52	37.72	39.2	45.33	34.17	36.95	37.02	49.13	60.11	34.17	81.11	51.4±16.55
	1o	78.8	77.72	60.75	41.26	36	37.7	43.92	44.49	43.4	30.91	43.21	59.05	30.91	78.8	49.77±15.78
	2i	80.89	85.22	70.85	51.62	51.8	47.51	49.7	49.04	47.11	46.22	49.61	67.92	46.22	85.22	58.12±14.12
	2o	77.3	83.42	22.9	24.45	31.04	44.08	38.56	42.99	34.98	41.16	62.55	43.93	22.9	83.42	45.61±19.29
Slum area	1i	75.88	78.7	62.39	42.74	39.25	39	47.25	42.34	43.15	37.13	45.31	59.61	37.13	78.7	51.06±14.51
	1o	78.31	81.54	63.67	41.91	38.16	39.12	47.05	38.06	41.37	33.88	45.17	54.82	33.88	81.54	50.26±16.06
	2i	85.73	87.49	71.65	47.67	38.82	38.99	45.72	43.3	41.43	40.13	45.64	66.03	38.82	87.49	54.38±18.3
	2o	82.64	84.68	69.23	44.17	42.24	41.99	50.5	40.02	42.98	38.11	49.33	61.24	38.11	84.68	53.93±16.59
High-income Colony	1i	82.61	83	70.92	48.94	47.42	48.65	51.86	54.43	45.76	40.83	46.18	69.92	40.83	83	57.54±14.93
	1o	82.94	86.32	65.72	42.42	39.09	40.09	54.73	38.79	43.11	32.94	56.82	53.78	32.94	86.32	53.06±17.48
	2i	75.88	78.97	60.81	40.34	35.59	36.26	42.94	41.18	40.95	32.96	43.61	59.72	32.96	78.97	49.1±15.79
	2o	80.77	80.67	62.9	41.72	37.67	38.12	45.57	37.76	43.2	34.43	50.09	47.57	34.43	80.77	50.04±16.18
Middle-income Colony	1i	66.88	67.47	63.39	56.68	54.12	54.01	57.74	64.68	63.43	61.37	61.77	61.05	54.01	67.47	61.05±4.55
	1o	71.13	80.67	63.38	42.46	37.72	39.53	45.39	37.49	43.37	32.92	40.32	44.22	32.92	80.67	48.22±15.03
	2i	75.16	79.06	66.94	52.88	45.51	43.02	45.03	48.05	48.46	43.61	50.55	58.58	43.02	79.06	54.74±12.5
	2o	74.96	80.76	56.87	34.74	29	29.87	35.31	28.96	33.89	28.39	40.7	51.09	28.39	80.76	43.71±18.32
Low-income Colony	1i	77.75	81.44	70.46	50.83	47.21	46.99	53.9	50.76	48.89	43.12	49.21	58.95	43.12	81.44	56.63±12.84
	1o	76.82	79.99	62.52	43.59	44.16	42.65	51.26	38.01	40.82	32.32	33.74	56.38	32.32	79.99	50.19±15.82
	2i	72.2	76.89	62.51	43.69	42.79	40.61	43.82	44.24	42.35	34.89	43.92	54.82	34.89	76.89	50.23±13.38
	2o	75.41	78.1	63.24	44.45	41.51	40.96	46.82	42.75	42.44	34.92	45.86	54.21	34.92	78.1	50.89±14.04

Table S4. Monthly Month-wise Indoor CO_2 values at all sites (Where i=indoor, o=outdoor, 1=first house, 2=second house).

Location	Site	2022						2023								
		Jul	Aug	Sep	Oct	Nov	Dec	Jan	Feb	Mar	Apr	May	Jun	Minimum	Maximum	Mean ± Standard Deviation
Industrial area	1i	611.7	731.4	645	590	690.6	482.7	735	722	530.1	576.3	648.1	634.4	482.7	735	633.09±80.01
	1o	424.9	436.1	446.4	451	455.2	872.2	835	447.2	434.2	426.2	429.9	410.5	410.5	872.2	505.78±163.24
	2i	457.7	474	478.2	525	662.7	661.7	648	565.9	562.6	483.2	446.6	446	446	662.7	534.33±84.38
	2o	412.9	415.9	421.7	428	436.7	420.8	416	438.6	420.5	414.2	427.7	414.1	412.9	438.6	422.28±8.74
Commercial area	1i	507.9	530.4	511.4	539	580.4	574.5	512	453.3	462.9	526.7	515.3	604.3	453.3	604.3	526.48±44.46
	1o	417.8	432.7	420.8	446	463.4	465.4	512	638.4	494.9	425.4	425.3	421.8	417.8	638.4	463.68±63.08

Location	Site	Jul	Aug	Sep	Oct	Nov	Dec	Jan	Feb	Mar	Apr	May	Jun	Minimum	Maximum	Mean ± Standard Deviation
	2i	470.3	476.4					####	740.5	603.6	536.6	537.3	651.1	470.3	4931.2	1118.38±1543.28
	2o	419.8	520			478.5	417.8	437	424.1	422.7	425.3	423.1	450.5	417.8	520	441.84±33.21
Slum area	1i	454.1	464.8	468.5	496	518.6	534.3	549	583.3	538.6	494.4	472.8	493.1	454.1	583.3	505.59±39.49
	1o	426.2	423.9	418.2	443	463.4	445.3	436	458	434.3	423	432.1	420.2	418.2	463.4	435.23±14.62
	2i	416.9	475.6	441.8	456	487.9	545.6	467	574.8	483	455.9	447.5	471.3	416.9	574.8	476.93±43.88
	2o	414.8	420.7	425.9	449	477.5	435	426	461	431.8	424.5	426.6	416.6	414.8	477.5	434.08±19.02
High-income Colony	1i	475	522.2	512.4	703	1173	1046	926	1280	982.6	685.9	437.4	466.9	437.4	1280	767.47±301.05
	1o	402.6	409	410.7	419	429.6	468.3	884	432.4	414.3	408.6	489.9	452.1	402.6	883.9	468.37±133.59
	2i	433	444.9	443.9	476	508.5	548.3	587	570.3	561.3	481.3	494.8	442	433	586.9	499.26±55.3
	2o	405.6	410.8	410	425	439.9	422.2	412	437.4	422.4	419.1	420.5	412.9	405.6	439.9	419.81±10.68
Middle-income Colony	1i	1184	1461.8	1210	1294	1105	946.9	797	1047.1	970.5	1054.6	1027	1303.9	796.6	1461.8	1116.88±183.03
	1o	876.9	409.4	414.9	422	435.4	430.2	413	445.3	422.1	459.2	422.8	437.4	409.4	876.9	465.78±130.26
	2i	591.5	623.3	620.3	604	568.4	569.6	626	750.4	670.9	659.2	642.8	587	568.4	750.4	626.11±50.99
	2o	414.3	415.7	407.5	418	424.5	416.6	415	428.6	419.3	411.1	416	411.8	407.5	428.6	416.5± 5.72
Low-income Colony	1i	564.4	547.4	561.1	545	605.4	598.5	671	700.4	689.8	674.1	528.8	492.5	492.5	700.4	598.25±69.99
	1o	413	412	417.5	425	444.4	417.6	417	432.3	417.2	418.5	453.1	434.3	412	453.1	425.12±13.16
	2i	485.1	516.8	503.6	500	497.8	518.9	559	581.2	561.3	412.2	418.3	499.8	412.2	581.2	504.46±51.19
	2o	408.6	414.1	418.5	429	530.7	473.9	417	435	419.6	412.1	519.9	453.3	408.6	530.7	444.23±42.37

Table S5. Monthly Air Quality Index (AQI US) values at all sites (Where i=indoor, o=outdoor, 1=first house, 2=second house).

Location	Site	2022						2023								
		Jul	Aug	Sep	Oct	Nov	Dec	Jan	Feb	Mar	Apr	May	Jun	Minimum	Maximum	Mean ± Standard Deviation
Industrial area	1i	73.95	100.06	124.56	151.43	173.22	178	167.1	147.96	101.84	57.69	48.96	71.655	48.96	177.96	116.37 ±46.86
	1o	68.37	95.19	131.27	146.23	194.2	165.2	167.34	153.48	102.43	75.12	77.01	87.86	68.37	194.20	121.98 ±42.82
	2i	55.52	73.14	105.73	130.95	172.47	161.2	146.34	121	59.28	34.85	21.94	63.975	21.94	172.47	95.53 ±50.75
	2o	50.1	68.33	90	119.65	161.12	146	126.35	96.65	38.84	17.01	15.2	57.17	15.20	161.12	82.2 ±45.98
Commercial area	1i	49.17	67.07	95.58	107.5	161.13	160.7	87.49	18.1	28.44	81.64	71.81	86.475	18.10	161.13	84.59 ±44.32
	1o	58.04	79.84	98.03	121.47	104.31	134.9	72.98	145.66	49.89	3.58	5.19	53.75	3.58	145.66	77.30 ±46.16
	2i	57.85	82.04	97.17	146.95	222.43	154.6	186.59	150.64	122.99	109.1	91.38	101.17	57.85	222.43	126.91 ±47.04
	2o	52.49	67.49			141.22	161.7	142.37	103.52	91.32	96.93	71.36	98.395	52.49	161.67	102.68 ±35.68
Slum area	1i	82.71	94.92	136.99	192.81	229.87	221.5	208.41	212.87	153.16	125.8	96.88	109.02	82.71	229.87	155.41 ±54.91
	1o	78.35	100.09	127.55	172.15	193.92	156.6	164.71	163.99	121.52	137.7	116.71	118.85	78.35	193.92	137.67 ±33.33
	2i	76.69	88.72	117.72	150.39	186.78	184.3	187.14	183.79	145.29	106.8	112.69	112.86	76.69	187.14	137.77 ±40.55

	2o	83.42	103.31	129.2	156.72	201.09	186.6	183.52	153.95	112.84	94.51	92.3	114.37	83.42	201.09	134.32 ±40.78
High-income Colony	1i	57.71	68.91	86.48	98.42	128.58	128.5	140.98	114.12	63.56	55.87	70.58	79.15	55.87	140.98	91.07 ±30.25
	1o	58.22	77.38	92.3	115.53	160.61	151.7	137.63	135.59	90	76.52	59.5	93.945	58.22	160.61	104.07 ±35.31
	2i	53.28	62.18	79.04	101.66	141.49	131.5	140.77	135.52	89.26	65.94	62.79	90.87	53.28	141.49	96.2 ±33.39
	2o	62.29	84.65	101.26	125.24	171.87	165.2	167.68	142.36	95.25	91.42	85.1	123.15	62.29	171.87	117.96 ±37.02
Middle-income Colony	1i	64.47	94.41	89.65	97.06	112.13	143.5	157.56	158.47	116.34	87.3	75.23	73.225	64.47	158.47	105.78 ±32.39
	1o	69.05	78.21	93.47	122.56	162.79	162.6	163.45	144.51	99.4	79.44	76.1	109.62	69.05	163.45	113.43 ±36.65
	2i	93.68	101.94	100.01	129.34	152.19	148.8	158.73	124.45	88.99	77.63	80.56	100.83	77.63	158.73	113.09 ±28.6
	2o	50.38	69.42	75.95	109.39	151.62	151.7	158.77	134.32	88.5	67.12	59.64	79.33	50.38	158.77	99.68 ±39.66
Low-income Colony	1i	70.41	100.67	103.23	119.13	156.56	150.5	161.35	145.53	122.76	94.77	68.96	67.99	67.99	161.35	113.49 ±34.63
	1o	44.09	55.12	101.68	113.21	156.91	154.6	158.95	133.49	99.2	80.78	73.62	93.26	44.09	158.95	105.41 ±39.18
	2i	36.74	45.74	75	106.09	155.53	144.8	138.38	134.67	84.32	43.63	55.38	47.36	36.74	155.53	88.97 ±44.82
	2o	38	49.31	74.74	104.93	156.79	144.5	140.51	113.82	69.76	43.58	43.04	57.82	38.00	156.79	86.40 ±43.71

**Air Quality Index (AQI)**	0-50	Good	51-100	Satisfactory	101-200	Moderate	201-300	Poor	301-400	Very Poor	401-500	Severe

Table S6. Microbial counts in November 2022.

S.No.	Location	Area	Bacterial colony count (CFU/plate)	Fungal colony count (RB-PDA) (CFU/plate)
1.	**Industrial area**	Living area	416	28
		Outdoor	157	12
		Kitchen	201	19
		Bedroom	181	32
		Bathroom	263	25
2.	**High income colony**	Living area	221	31
		Outdoor	283	38
		Kitchen	117	24
		Bedroom	189	22
		Bathroom	99	23
3.	**Slum arera**	Living area	385	71
		Outdoor	348	82
		Kitchen	269	45
		Bedroom	307	49
		Bathroom	282	82
4.	**Commercial area**	Living area	251	65
		Outdoor	289	92
		Kitchen	165	113
		Bedroom	280	56
		Bathroom	186	73
5.	**Middle income colony**	Living area	169	30
		Outdoor	182	37
		Kitchen	149	27
		Bedroom	117	17
		Bathroom	274	27
6.	**Low income colony**	Living area	54	11

		Area	Bacterial colony count	Fungal colony count
		Outdoor	76	29
		Kitchen	56	17
		Bedroom	31	10
		Bathroom	68	25

Table S7. Microbial counts in February 2023.

S.No.	Location	Area	Bacterial colony count (CFU/plate)	Fungal colony count (RB-PDA) (CFU/plate)
1.	**Industrial area**	Living area	428	21
		Outdoor	576	26
		Kitchen	388	28
		Bedroom	336	15
		Bathroom	404	33
2.	**High income colony**	Living area	76	19
		Outdoor	216	32
		Kitchen	103	17
		Bedroom	51	20
		Bathroom	68	23
3.	**Slum arera**	Living area	336	61
		Outdoor	364	81
		Kitchen	288	55
		Bedroom	344	39
		Bathroom	284	42
4.	**Commercial area**	Living area	348	21
		Outdoor	366	49
		Kitchen	472	35
		Bedroom	224	24
		Bathroom	316	25
5.	**Middle income colony**	Living area	216	9
		Outdoor	392	41
		Kitchen	192	10
		Bedroom	152	12
		Bathroom	132	23
6.	**Low income colony**	Living area	127	76
		Outdoor	248	128
		Kitchen	178	76
		Bedroom	152	83
		Bathroom	488	89

Table S8. Microbial counts in May 2023.

S.No.	Location	Area	Bacterial colony count (CFU/plate)	Fungal colony count (RB-PDA) (CFU/plate)
1.	**Industrial area**	Living area	76	8
		Outdoor	193	1
		Kitchen	63	4

		Bedroom	32	0
		Bathroom	51	3
2.	**High income colony**	Living area	137	8
		Outdoor	208	26
		Kitchen	166	9
		Bedroom	143	18
		Bathroom	152	9
3.	**Slum arera**	Living area	208	27
		Outdoor	264	26
		Kitchen	324	7
		Bedroom	166	18
		Bathroom	276	9
4.	**Commercial area**	Living area	163	19
		Outdoor	167	24
		Kitchen	112	12
		Bedroom	127	11
		Bathroom	118	8
5.	**Middle income colony**	Living area	280	6
		Outdoor	348	14
		Kitchen	292	5
		Bedroom	308	8
		Bathroom	362	2
6.	**Low income colony**	Living area	126	3
		Outdoor	264	15
		Kitchen	187	5
		Bedroom	106	7
		Bathroom	144	6

Table S9. Microbial counts in July 2023.

S. No.	Location	Area	Bacterial colony count (CFU/plate)	Fungal colony count (RB-PDA) (CFU/plate)
1.	**Industrial area**	Living area	TNTC	9
		Outdoor	TNTC	21
		Kitchen	TNTC	15
		Bedroom	TNTC	13
		Bathroom	TNTC	12
2.	**High income colony**	Living area	TNTC	8
		Outdoor	TNTC	19
		Kitchen	TNTC	18
		Bedroom	TNTC	13
		Bathroom	TNTC	12
3.	**Slum arera**	Living area	TNTC	36
		Outdoor	TNTC	57
		Kitchen	TNTC	18
		Bedroom	TNTC	31
		Bathroom	TNTC	53
4.	**Commercial area**	Living area	TNTC	69
		Outdoor	TNTC	65
		Kitchen	TNTC	28
		Bedroom	186	28

		Bathroom	TNTC	17
5.	**Middle income colony**	Living area	192	7
		Outdoor	TNTC	22
		Kitchen	TNTC	7
		Bedroom	TNTC	16
		Bathroom	TNTC	5
6.	**Low income colony**	Living area	TNTC	7
		Outdoor	TNTC	18
		Kitchen	160	12
		Bedroom	TNTC	3
		Bathroom	216	7

*TNTC- too numerous to count
